# Supplementary material for: Association of HIV infection and antiretroviral therapy with the occurrence of an unfavorable TB treatment outcome in a rural district hospital in Eastern Cape, South Africa: A retrospective cohort study
Source: PLoS One. 2022 Apr 5;17(4):e0266082. doi: 10.1371/journal.pone.0266082 (PMC8982869; doi:10.1371/journal.pone.0266082)
Supplement: S1 File — Investigating missing outcomes. (DOCX) [file pone.0266082.s001.docx]

**S1 File. Investigating missing outcomes**

295 (29%) of the initial patients diagnosed with TB at a district hospital had undocumented outcomes and/or missing HIV/ART category. As described in the main text, we excluded these individuals from the main analysis under the assumption that they were missing completely at random. In Table S1, we compare key demographics of patients by those excluded due to missing values (N=295) and included for complete case analysis (N=711). We only find significant differences in the TB Type variable.

As a sensitivity analysis, we perform missing imputation by chained equations using the mice package in R. We created 20 datasets with imputed missing values across all variables (including covariates such as alcohol use). The variables included in the imputation were those listed in Table 1, including the treatment outcome. Table S2 (analogue of Table 3) show the pooled results from the imputed datasets.

**Table S1.** **Key demographics of patients diagnosed with TB at a district hospital by inclusion in main analysis (N=1006)**

|  | **Excluded**  **(N=295)** | **Included**  **(N=711)** | **P-value^1^** |
| --- | --- | --- | --- |
| **Age** *Median (IQR)* | 39 (30, 59) | 38 (30, 57) | 0.298 |
| **Male Gender** | 149 (50.7) | 355 (50.0) | 0.899 |
| **HIV/ART Category** | | | |
| HIV+, no ART | 39 (17.8) | 104 (14.6) | 0.152 |
| HIV+, ART | 81 (37.0) | 314 (44.2) |  |
| HIV- | 99 (45.2) | 293 (41.2) |  |
| **Tobacco Use** | | | |
| No | 170 (57.6) | 405 (57.0) | 0.758 |
| Yes | 36 (12.2) | 99 (13.9) |  |
| *Unanswered* | *89 (30.2)* | *207 (29.1)* |  |
| **Alcohol Use** | | | |
| No | 167 (56.6) | 386 (54.3) | 0.595 |
| Yes | 40 (13.6) | 114 (16.0) |  |
| *Unanswered* | *88 (29.8)* | *211 (29.7)* |  |
| **TB Type** | | | |
| Pulmonary | 149 (50.5) | 441 (62.0) | 0.004 |
| Extra Pulmonary | 111 (37.6) | 213 (30.0) |  |
| Both | 15 (5.1) | 31 (4.4) |  |
| *Unanswered* | *20 (6.8)* | *26 (3.7)* |  |

^1^ Wilcoxon rank sum (age) and Fisher’s exact test were used to test for differences in distribution by HIV/ART category. Unanswered values were excluded from testing.

**Table S2.** **Estimated odds ratios for Down Referral vs. Unfavorable Treatment Outcome and Success vs. Unfavorable TB Treatment Outcome (N=1006)**

|  | Estimated OR for Down referral vs. Unfavorable TB Treatment Outcome | | Estimated OR for Successful vs. Unfavorable TB Treatment Outcome | |
| --- | --- | --- | --- | --- |
| Variable | Odds Ratio and 95% CI | p-value | Odds Ratio and 95% CI | p-value |
| HIV+, no ART | 1 (Reference) |  | 1 (Reference) |  |
| HIV+, ART | 1.6 (0.87, 2.94) | 0.13 | 2.17 (1.12, 4.22) | 0.023 |
| HIV- | 2.99 (1.53, 5.86) | 0.002 | 4.75 (2.24, 10.08) | 0 |
| Age category | | | | |
| 15-24 | 1 (Reference) |  | 1 (Reference) |  |
| 25-54 | 0.83 (0.39, 1.76) | 0.633 | 0.88 (0.39, 1.98) | 0.754 |
| 55-64 | 0.48 (0.2, 1.15) | 0.101 | 0.41 (0.16, 1.04) | 0.062 |
| 65+ | 0.22 (0.09, 0.5) | 0 | 0.27 (0.11, 0.66) | 0.004 |
| Sex | | | | |
| Female | 1 (Reference) |  | 1 (Reference) |  |
| Male | 0.96 (0.57, 1.61) | 0.874 | 0.97 (0.57, 1.66) | 0.924 |
| TB Type | | | | |
| Pulmonary | 1 (Reference) |  | 1 (Reference) |  |
| Extra Pulmonary | 0.62 (0.36, 1.08) | 0.093 | 1.3 (0.78, 2.14) | 0.314 |
| Both | 0.28 (0.1, 0.8) | 0.018 | 0.81 (0.32, 2.07) | 0.662 |
| Alcohol use | | | | |
| Yes | 1 (Reference) |  | 1 (Reference) |  |
| No | 1.93 (0.78, 4.78) | 0.163 | 1.64 (0.61, 4.42) | 0.332 |
| Tobacco use | | | | |
| Yes | 1 (Reference) |  | 1 (Reference) |  |
| No | 0.41 (0.17, 1) | 0.055 | 0.38 (0.16, 0.91) | 0.034 |
