# Supplementary material for: Association of HIV infection and antiretroviral therapy with the occurrence of an unfavorable TB treatment outcome in a rural district hospital in Eastern Cape, South Africa: A retrospective cohort study
Source: PLoS One. 2022 Apr 5;17(4):e0266082. doi: 10.1371/journal.pone.0266082 (PMC8982869; doi:10.1371/journal.pone.0266082)
Supplement: Attachment — Submitted filename: Response to reviewers_PLOSONE_TBtxoutcomes_10192021.docx [file pone.0266082.s002.docx]

RE: PLOS ONE PONE-D-21-14796

Dear Dr. Balasubramani and reviewers,

Thank you very much for your thorough review and comments on our manuscript. We have revised the manuscript to be a much-improved version addressing all of your concerns.

Below we list how we have addressed each reviewer’s comment.

Ethics approval was provided by Walter Sisulu University in South Africa, the Eastern Cape Department of Health, and the Harvard Medical School Institutional Review Board.

Thank you for the opportunity to revise our manuscript. We look forward to hearing back soon.

Sincerely,

Brittney van de Water, on behalf of the authors

**Editors Comments**

**Editor 1.1**: Please ensure that your manuscript meets PLOS ONE's style requirements, including those for file naming.

RESPONSE: Thank you. We have reviewed the style requirements and have named files appropriately.

Editor 1.2: In your ethics statement in the Methods section and in the online submission form, please provide additional information about the data used in your retrospective study. Specifically, please ensure that you have discussed whether all data were fully anonymized before you accessed them and/or whether the IRB or ethics committee waived the requirement for informed consent. If patients provided informed written consent to have data from their medical records used in research, please include this information.

RESPONSE: Thank you. We have added into our Ethics statement “Data used in this retrospective study were identifiable upon collection; however, were fully deidentified and anonymized before statistical analysis. Both IRBs waived informed consent.”.

Editor 1.3: We note that you have indicated that data from this study are available upon request. PLOS only allows data to be available upon request if there are legal or ethical restrictions on sharing data publicly.

RESPONSE: Thank you for this guidance. We will make a limited dataset available through Boston College’s Dataverse repository.

Editor 1.4: In your revised cover letter, please address the following prompts:

a) If there are ethical or legal restrictions on sharing a de-identified data set, please explain them in detail (e.g., data contain potentially sensitive information, data are owned by a third-party organization, etc.) and who has imposed them (e.g., an ethics committee). Please also provide contact information for a data access committee, ethics committee, or other institutional body to which data requests may be sent.

RESPONSE: Thank you. As noted above, we have no limitations and data will be deposited into Boston College’s Dataverse repository.

b) If there are no restrictions, please upload the minimal anonymized data set necessary to replicate your study findings as either Supporting Information files or to a stable, public repository and provide us with the relevant URLs, DOIs, or accession numbers. For a list of acceptable repositories, please see: <http://journals.plos.org/plosone/s/data-availability#loc-recommended-repositories>. We will update your Data Availability statement on your behalf to reflect the information you provide.

RESPONSE: Many thanks.

Editor 1.4: Please include a separate caption for each figure in your manuscript.

RESPONSE: Thank you – we have added a separate caption for Figure 1 (and have omitted Figure 2 in line with reviewers’ comments).

Editor 1.5: Please ensure that you refer to Figure 1 in your text as, if accepted, production will need this reference to link the reader to the figure.

RESPONSE: Thank you, and apologies for omitting. We have included reference to Figure 1 in the first paragraph of the Methods section.

**Reviewer 1 Comments**

Reviewer 1.1: There is no mention about the confounding effect to type of TB which could have influence on the outcome and on HIV treatment. It is not clear if this is considered in the current analysis. Also, other covariates such as alcohol and tobacco use may also have an influence on the TB outcome.

RESPONSE: Thank you for this comment. We have now adjusted for TB type, alcohol use, and tobacco use in the analysis with minimal change, as detailed in the Methods, Statistical Analysis section.

Reviewer 1.2: Although ORs and CIs are for the main analysis are provided, some additional details on original parameter estimates, SE and the p-values for the HIV status variable and for the covariates in the model would be useful. Results in table 3 and 4 can be provided together as these are from single multinomial logit analysis.

RESPONSE: Thank you. We have added p-values to tables 3 and 4 and have combined them into just one table, Table 3. Note that we do not report standard errors as these are less interpretable on the odds ratio scale.

Reviewer 1.3: The authors should briefly discuss the type of TB treatment which could be useful for the reader interested in TB research.

RESPONSE: Thank you. We have added “The vast majority of adults ≥ 15 years with drug sensitive TB in South Africa is managed with four drugs for the first two months of the “intensive phase“ of treatment: rifampicin, isoniazid, pyrazinamide and ethambutol (RHZE), followed by four months of rifampicin and isoniazid (RH) – the “continuation phase”, as per the South African National TB guidelines.“ into the Methods section for background on types of TB treatments used during this period in South Africa.

Reviewer 1.4: Table 1 has lot of additional information about covariates/illness but the details about the assessment of these conditions (whether self-reported or diagnosed) are missing. Separate row for p-value in this table can be omitted. Are these reported p-values for three group or two group comparisons? It is not easy to understand this as the authors discuss the results separately for HIV treatment groups.

RESPONSE: Thank you for this comment. We added in the Methods, Data sources and study measures section that “Baseline demographic and medical history were collected through self-report and an interview”. To clarify, we have added into the Methods that p-values correspond to a Fisher’s exact test that compares across all three groups simultaneously (HIV negative, HIV positive on ART, and HIV not on ART). This can be interpreted as testing for differences in the distribution of HIV/ART for each demographic characteristic.

Reviewer 1.5: Figure 1 is important for the reader to understand the numbers presented in the study design is not mentioned inside the text.

RESPONSE: Thank you for noticing this. We have now referenced Figure 1 in the text at first mention in the Methods section, Study design and study population.

Reviewer 1.6: Does “unanswered” refer to missing data or refused to answer (if self-reported)?

RESPONSE: Thank you for this question. As described in the Methods, “unanswered” refers to questions that were typically not asked by the clinician. In trying to not “patient blame” we want readers to understand that often it is not because patients are non-forthcoming with their health status, rather often times it is the provider (nurse or doctor) who never asks the question and/or leaves it blank. Of note, these charts are from a low-resourced setting where clinicians are often hard pressed for time and charting is not always the priority. The sentence reads “Missing values for covariates were given their own category of “Missing”, often when a clinician did not ask or did not tick “yes” or “no” in the medical chart”.

Reviewer 1.7: It is not clear why the authors present the age results with 936 samples when all other results are based on 711 samples.

RESPONSE: Thank you for this comment. Our original analysis only focused on adults, but we chose to display results for individuals <15 years in Figure 2. We now realize that this caused confusion and have removed persons <15 years from Figure 2 and only focus on adults in this manuscript and Figure 2.

Reviewer 1.8: Incidence and prevalence estimates should have a time interval.

RESPONSE: Thank you for this concern. We have added dates for prevalence and incidence referenced in the Methods per the cited references.

Reviewer 1.9: It is hard to follow the description provided in data source and study measures. The authors should describe the information about some of the self-reported measures.

RESPONSE: Thank you for this comment. We have revised the Methods section which now reads “Baseline demographic and medical history were collected through self-report and an interview with a healthcare provider as the TB file was started…” and expands on self-reported measures.

Reviewer 1.10: Provide the reference for R software and the r-function used for the multinomial logit model.

RESPONSE: Thank you for catching this. We have now included the R software and R-function used for analysis in the Methods. We also include the R scripts necessary to run our analyses.

**Reviewer 2 Comments**

Reviewer 2.1: A major limitation of this paper is the number of people excluded in this study (414 no treatment outcome, 77 no HIV status reported and 212 < 15 years). They start with 1429 subjects with TB and the analysis sample contains only 711 (48%). They claim that missing completely was at random but no results are present to substantiate this claim.

RESPONSE: We appreciate your concern. We have revised the Methods to only describe individuals who were > 15 years. Therefore, it now reads “Data for this study were taken from TB medical charts of a cohort of individuals 15 years or older who were diagnosed with drug-sensitive TB and who should have completed treatment at the district hospital between January 4, 2017 and April 27, 2020 (N=1006) (Fig 1). There were 295 individuals excluded due to no documented treatment outcome (n=246) or no HIV status reported (n=49) for a final sample size of 711.” We also added into the Data Sources/Study Measures section “Missing values for covariates were given their own category of “Missing”, often when a clinician did not ask or did not tick “yes” or “no” in the medical chart, as described above, this is a busy hospital with busy clinicians and lacks administrative capacity to close loops for charting, which does not impact clinical care. For missing HIV status, often it was due to tests not being available on the day a patient was seen, and then no HIV status was recorded upon testing on a subsequent day after the initial baseline data collection. Finally, a final treatment outcome is not always recorded by clinicians in the medical chart. Therefore, we believe these missing data points are missing at random.”. Also, we have now included a supplemental analysis where we (1) investigate differences in demographic characteristics between our included study population and those that were excluded due to missingness and (2) repeated analysis with a multiple imputation procedure to impute the missing outcomes and HIV status. Importantly, we feel confident that our results were not impacted by this large source of missingness as we found minimal differences in demographic characteristics and the results did not change in our imputed version.

Reviewer 2.2: Lines 102 -106 talks about children ≤ 15 years that are excluded from the study and not appropriate in the methods section.

RESPONSE: Thank you. We have removed the sentences regarding the rationale and only provide inclusion/exclusion criteria now. We have also cited 3 additional sources explaining why we excluded individuals ≤ 15 years.

Reviewer 2.3: Line 111 - The TB incidence rate is mentioned as 838/100,000 per year. If this is the case, then wouldn't we expect much more cases during the study period (Jan 2017 to Apr 2020)? (even after excluding children). Were all TB cases in the Eastern Cape accounted in this study?

RESPONSE: Thank you for this observation. No, not all cases of TB were accounted for in this study – this study was only at one district hospital in a rural area with a population of nearly 130,000. There are 12 referring clinics to the hospital where many cases of TB are also diagnosed. We only provided this information as background information about the setting.

Reviewer 2.4: Line 117 Is the adult unemployment population 91.5%? This appears to be very high. (9.9% reported mining work alone!)

RESPONSE: Thank you. Reviewing the data we took from the official 2011 South African Census (as presented through Wazimap.co.za), we realize that the manner in which we have reported this data may be unclear. Although the rate of employment in the catchment area of Zithulele Hospital, at 9.7% of working-age adults is accurate and indeed exceedingly low, the remainder of the adults should not, strictly speaking, be categorized as “unemployed”. The South African Census data uses the following categories and definitions:

**Employed:** Persons who worked for pay, profit, or family gain, even for just one hour, in the seven days before they were interviewed.

**Unemployed (official definition):** Persons who did not work, but who looked for work and were available to work in the past seven days.

**Discouraged work-seeker:** Someone who has lost hope in finding any kind of work.

**Not economically active:** Persons who were neither employed nor unemployed (e.g. full-time students; retired persons; and homemakers who did not want to work).

Looking at the areas/wards making up the catchment area of Zithulele Hospital (excluding Mthatha, a major city in KSD sub-district), the percentages for each category are as follows:

Employed: 9.7%

Unemployed: 9.6%

Discouraged work seeker: 10.5%

Not economically active: 70.2%

It is therefore more accurate to report an employment rate of 9.7%, an unemployment rate of 20% (of which half are discouraged work seekers) and an extremely high proportion (70.2%) of economically inactive people - which we have now corrected in the manuscript.

We have also changed the Setting section to now read “The median annual household income is R14600 (US$1000)”.

Reviewer 2.5: Line 124 Why was the rapid cartridge HIV antibody test not done for the 77 patients with no HIV status?

RESPONSE: Thank you for this astute observation. As with all types of clinical care in low resource settings, not all evaluations and tests are done for every individual. At times the hospital runs out of cartridges, clinicians are too rushed to do an examination at the initial visit and plan to do it later, or in most cases the rapid test was completed, and just not documented in the source document. Three co-authors are clinicians in the hospital and confirm that due to extenuating circumstances, things do get missed from time to time.

Reviewer 2.6: Line 129-130 Who created the composite categorical outcomes? Why couldn't it be created for the 414 patients?

RESPONSE: Thank you. We (BvdW, IF, and KlR) created the composite categorical outcomes. These were created from source document outcomes including: loss to follow-up, death, treatment failure, cure, completed treatment, and down referral. Those 414 individuals did not have a source document outcome documented. Similar to the above response regarding missing HIV status, clinicians do not always document a final outcome in the TB Medical Record. We have now included a Supplement with an investigation into the missing data. Please see our response to Comment 2.1.

Reviewer 2.7: Line 153 - Why was the "unfavorable" outcome chosen as the reference category (which includes loss to follow-up)? Why can't the majority (88%) who had positive outcomes be the reference category?

RESPONSE: Thank you. We chose “unfavorable” as the reference category because both “success” and “down referral” are considered “positive” outcomes, so it did not make sense to do a comparison of “success” to “down referral” but rather compare each of these to “unfavorable”. Importantly, this was the reference category preferred by our collaborators at the hospital, and we agree that it is easier to interpret “positive” outcomes vs. “negative” outcomes in this manner. Lastly, the reference category does not change the results and an interested reader has all the available information to “flip” the reference category if they so wished.

Reviewer 2.8: Line 155- Why can't age be introduced as a continuous variable in the models? Dichotomizing a continuous variable arbitrarily with a cut-off as 45 years may not be appropriate. Is there any clinical evidence? Would childbearing age be appropriate for males too?

RESPONSE: Thank you for this question. We chose not to include age as a continuous (linear) term because this would force the relationship between age and treatment outcome to be linear, which it is not. However, we agree with the Reviewer that this binary cutoff at 45 years is likely not sufficient. Our remaining options were to model age: (1) using a spline or polynomial terms or (2) with more categories informed from prior literature or our data. We chose the latter as this is more common in the literature, and we believe it accurately captures the non-linear relationship between age and the treatment outcome and is similar to what the recent South African Prevalence Survey had with age brackets. (Figure 2).

Reviewer 2.9: Table 1: Approximately 30% of variables in Table 1 appear to be missing (unanswered) and are excluded from tests of association. Are these variables self-reported or taken from the clinical notes?

RESPONSE: Thank you. Yes, we agree that many of the variables have high rates of missingness, hence, why we reported the amount “unanswered” in the table for transparency. This study was a retrospective chart review, and therefore all variables were taken from clinical charts (where they are primarily self-reported as described in the Methods).

Reviewer 2.10: Tables 2 and 3 are not presented in a logical manner and need to be summarized properly.

RESPONSE: Thank you for this helpful comment. We have combined Tables 3 and 4 into one Table, per other Reviewer comments, and additionally, to address your concern here, we have rearranged our Results to discuss down referral prior to successful outcomes.

Reviewer 2.11: Line 206 - Why are all age reported (n=936) when the study includes only >= 15 years (n=711). They are not relevant to the study.

RESPONSE: Thank you. We have omitted this paragraph to help streamline the paper and now only focus on adults (along with Figure 2).

Reviewer 2.12: Lines 218 - 223 The statements are ambiguous and not clear. Especially lines 222 & 223 are vague.

RESPONSE: Thank you for this comment. We have deleted those three sentences for clarity.

Reviewer 2.13: Line 274 - It is mentioned that 341 (34%) are excluded because of missing treatment outcomes. This is not in agreement with (n=414) presented in figure 1.

RESPONSE: Thank you. Apologies, it was a mistake that we mentioned n=341. We have edited the Methods to say “Data for this study were taken from TB medical charts of a cohort of individuals 15 years or older who were diagnosed with drug-sensitive TB and who should have completed treatment at the district hospital between January 4, 2017 and April 27, 2020 (N=1006) (Fig 1). There were 295 individuals excluded due to no documented treatment outcome (n=246) or no HIV status reported (n=49) for a final sample size of 711.”

Reviewer 2.14: Figure 2 should include only the study participants and not excluded children.

RESPONSE: Thank you – we have revised Figure 2.
